# Supplementary material for: Long distance multiplexed quantum teleportation from a telecom photon to a solid-state qubit
Source: Nat Commun. 2023 Apr 5;14:1889. doi: 10.1038/s41467-023-37518-5 (PMC10076279; doi:10.1038/s41467-023-37518-5)
Supplement: Supplementary file 1 — Supplementary Information [file 41467_2023_37518_MOESM1_ESM.pdf]

Supplementary Information for:
Long distance multiplexed quantum teleportation from a telecom photon to a solid-state qubit

Dario Lago-Rivera,[1] Jelena V. Rakonjac,[1] Samuele Grandi,[1] and Hugues de Riedmatten[1,2]
[1]*ICFO-Institut de Ciencies Fotoniques, The Barcelona Institute of Technology, Mediterranean Technology Park, 08860 Castelldefels (Barcelona), Spain*
[2]*ICREA-Institució Catalana de Recerca i Estudis Avançats, 08015 Barcelona, Spain*

In this Supplementary Information we:

- Introduce the theoretical explanation of our particular implementation of the quantum teleportation protocol: Supplementary note 1.
- Give further details about our experimental implementation, including the source of entangled photons, the qubit source, the Bell-state measurement, the quantum memory and the phase shifting: Supplementary note 2.
- Discuss the limitations on the measured fidelities: Supplementary note 3.

Supplementary note 1 TELEPORTATION OUTCOME

We use the time bin basis to encode the quantum states used in this experiment. Two time bins separated by 420 ns will be defined as our early $|e\rangle$ and late $|l\rangle$ bins.In order to perform our teleportation experiment two basic resources are needed:

- **Entangled state:** Our photon pair source naturally generates energy-time entangled photons. We pump it in continuous wave and a photon pair can be created in coherent superposition of all creation times within the coherence time of the pump laser. In order to make it compatible with the time bin basis that we use as encoding, we gate our heralding detectors such that we only accept events during the specific temporal bins that we defined as $|e\rangle$ and $|l\rangle$. In this way, we effectively have a source that produces the entangled state $|\Phi^+_{1,2}\rangle = 1/\sqrt{2}\,(|e_1e_2\rangle + |l_1l_2\rangle)$ between idler and signal photons.
- **Input qubit:** By modulating the amplitude and phase of attenuated laser light, we can arbitrarily generate qubit states in the time basis. Therefore we will in general be able to teleport the state $|\phi_3\rangle = \alpha\,|e\rangle + \beta\,|l\rangle$.

We can now rewrite the joint state of $|\Phi^+_{1,2}\rangle$ and $|\phi_3\rangle$ and derive the following expression:

$$|\Phi^+_{1,2}\rangle\otimes|\phi_3\rangle = \frac{1}{2}\left[|\Phi^+_{2,3}\rangle(\alpha\,|e_1\rangle + \beta\,|l_1\rangle) + |\Phi^-_{2,3}\rangle(\alpha\,|e_1\rangle - \beta\,|l_1\rangle) + |\Psi^+_{2,3}\rangle(\beta\,|e_1\rangle + \alpha\,|l_1\rangle) + |\Psi^-_{2,3}\rangle(\beta\,|e_1\rangle - \alpha\,|l_1\rangle)\right] \quad (1)$$

with the Bell States $|\Phi^\pm_{2,3}\rangle = 1/\sqrt{2}\,(|e_2e_3\rangle \pm |l_2l_3\rangle)$ and $|\Psi^\pm_{2,3}\rangle = 1/\sqrt{2}\,(|e_2l_3\rangle \pm |l_2e_3\rangle)$.

Therefore, if we project the joint state of the modes *2* and *3* into a Bell-state, we teleport the state initially encoded in the photonic mode *3* to the state of the photonic mode *1* with the only difference of a bit flip and/or a phase flip. The procedure used for measuring the Bell-state will be explained in Supplementary note 2 D.

Supplementary note 2 DETAILED SET-UP

In this section we are going to explain the details of the experimental set-up that we used for this experiment. The explanation will use supplementary figure 1 as a reference. Further details and characterizations not specific to this work can be found in previous works from our group at [1–3].

A Entanglement source

Our source is based on cavity-enhanced spontaneous parametric down conversion (cSPDC). A periodically poled lithium niobate (PPLN) crystal is placed inside an optical cavity which enhances the SPDC process within the cavity

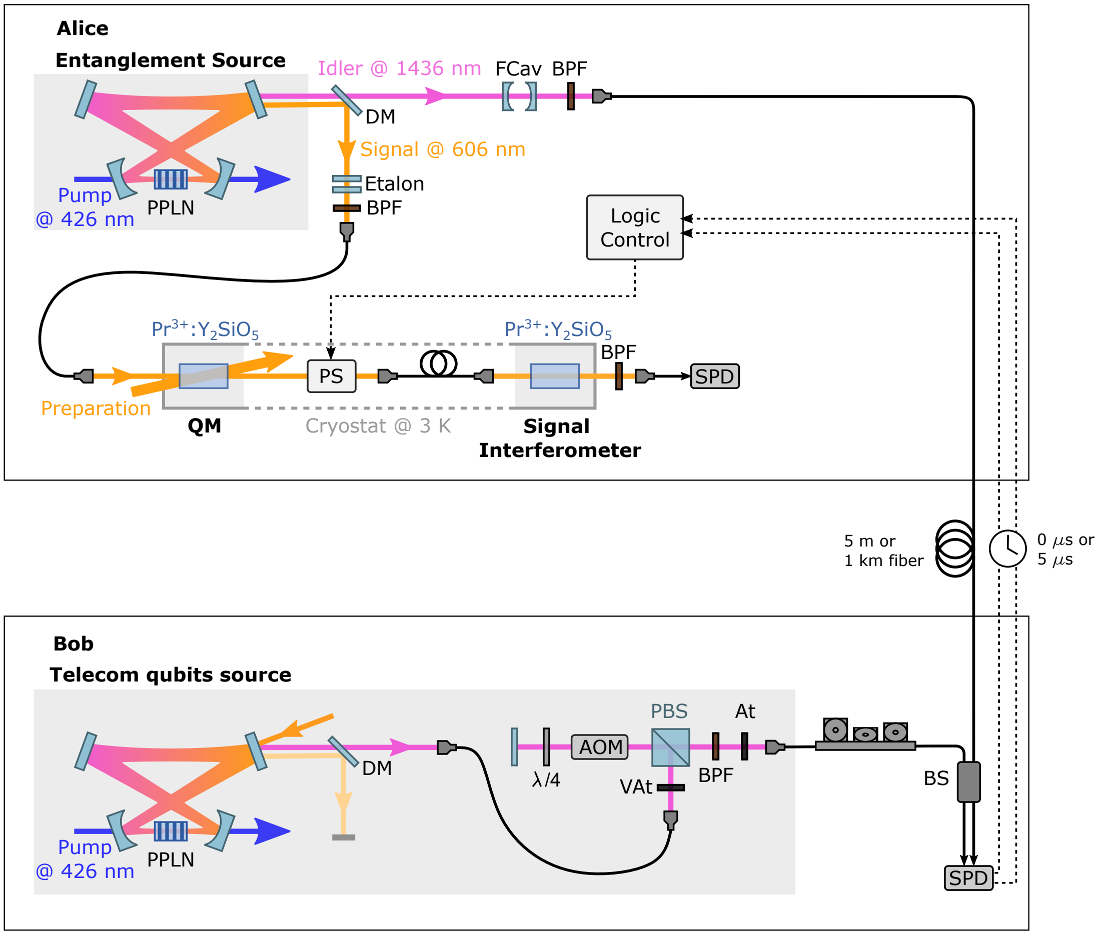

Supplementary figure 1. Detailed set-up: At Alice there is the entanglement source and the quantum memory while at Bob there is a telecom qubit source and the BSM set-up. The distance between Alice and Bon can be either a few meters or 1 km. List of abbreviations: DM - dichroic mirror, BPF - band pass filter, At - attenuator, VAt - variable attenuator, AOM - acousto optical modulator, SPD - single photon detector, PBS - polarizing beam splitter, FCav - filter cavity, PPLN - periodically-poled lithium niobate, PS - phase shifer, QM - quantum memory, BS - beam splitter.

modes. Consequently, only frequencies compatible with the cavity modes are generated. Pumping the source with a continuous wave laser at 426 nm results in the generation of an idler photon at 1436 nm of wavelength, corresponding to the telecom E-band and a signal photon at 606 nm, compatible with storage in our Pr^{3+} quantum memory (QM). We add spectral filters in order to ensure single frequency mode operation [4] and reduce broad-band noise. The filters include a Fabry-Perot cavity (FCav) with a linewidth of 80 MHz in resonance with the central idler frequency mode and an etalon filter with a linewidth of 4 GHz in the signal photons path. Note that in the case of the 606 nm photons, the AFC stores the central frequency mode, while the inhomogeneous broadening of the Pr^{3+} doped crystal, spanning a range of around 10 GHz, absorbs the extra frequency modes, such that the QM effectively acts as a narrow filter. In addition, we use band-pass filters (BPF) for a final broadband filtering before the single photon detectors (SPD). A more detailed discussion about the spectral characterization of the cSPDC source can be found in [1, 5, 6].

In order to create energy-time entanglement the coherence time of the pump laser has to be much larger than the coherence time of the photon pair, i.e. $\tau_{pump} \gg \tau_{pair}$. While τ_{pair} is fixed by the cavity linewidth as 120 ns, the coherence time of the free-running pump laser (TOPTICA TA - SHG 110) does not fulfill this condition. To increase it, we lock the pump laser to a reference cavity that allows us to reach $\tau_{pump} \approx 1$ μs.

We send the signal photon to the QM set-up to store it and we forward the idler telecom photon to the Bell-state measurement station at Bob's side by coupling it into a single mode optical fibre that can be either a short fibre (5 m) or a spool of 1 km.

Supplementary table 1. Table with the frequency shifts involved in the state generation to ensure indistinguishability at the BSM set-up.

	Entanglement source	Qubits source
Pump	ν_{426nm}	$\nu_{426nm} - 110\ MHz$
Ref 606 nm - Seed	ν_{606nm}	$\nu_{606nm} - 320\ MHz$
Telecom light at the BSM	ν_{1436nm}	$\nu'_{1436nm} + 210\ MHz$

B Telecom qubit source

Indistinguishability between the idler photon and the input qubit at the beam splitter is a fundamental requirement for a succesful BSM. For this purpose, we had to produce qubits at the same wavelength as the idler photons, i.e. 1436 nm, with sub-MHz precision. To do so, we used a second PPLN crystal embedded inside another optical cavity operated as an optical parametric oscillator (OPO). By seeding and pumping it with 606 nm and 426 nm of light respectively, we produced coherent light at 1436 nm. In addition, we used an acousto-optic modulator (AOM) in a double-pass configuration to move from the continuous wave light of the OPO to a pulsed operation. By properly modulating the amplitude of the pulses we could tailor their temporal profile to mimic that of the biphoton from the entanglement source. In general, two of these pulses separated by 420 ns form our input qubits $|\phi_3\rangle$. Note that the AOM also acts as a phase modulator that allows us to set the relative phase between the $|e\rangle$ and $|l\rangle$ time bins of the qubits. Finally, we used a combination of neutral density (ND) filters and a variable attenuator to bring the mean photon number per qubit to the single photon level. For all the experiments presented in the main text we used a mean photon number of 0.02 at the beam splitter of the Bell-state measurement. We also removed broad-band noise using an additional BPF.

Note that as a consequence of using an AOM to modulate the OPO light, there will be an extra frequency shift on this telecom field. We have to ensure that the frequency of the qubits and the idler photons interfering at the BSM is the same. This is why we carefully shifted the frequency of the fields used in the OPO as well as in the AOM for generating the input qubits. In supplementary table 1 we write all the frequency shifts involved where energy conservation imposes the relation $\nu_{426nm} - \nu_{606nm} = \nu_{1436nm}$. Thanks to the specified shifts, we could ensure that $\nu_{1436nm} = \nu'_{1436nm}$.

C Experiment control

To control different aspects of the experimental setup, we use a series of modules from Signadyne (now part of Keysight), which can operate simultaneously and are synchronised through the use of the M3601A software (Keysight).

One module is used for arbitrary waveform generation (AWG), which is needed for the AFC preparation in memory and the qubit analyzer, as both require the use of particularly chosen optical pulses that are shaped by AOMs in a double-pass configuration. It is also used for the modulation of the AOM of the telecom qubit source (which is integral to the synchronisation of the Bell-state measurement), and the reference 606 nm light of the entanglement source (to ensure the 606 nm photons are generated at the same frequency of the AFC).

A time-to-digital converter (TDC) module is used to acquire photon detection events. The acquisition can be gated on or off in order to separate detection events into distinct time bins. We can also use this module to implement basic arithmetic and logical operations. This forms the basis of the logic control of our experiment; see Supplementary note 2 D for details.

Based on the outcome of the Bell-state measurement, we need to apply a phase shift (Supplementary note 2 F). To control the driver for the phase shifter, we use a digital input/output (DIO) module to send a TTL signal when it needs to be switched on. We also use this module to send triggers to the TDC card to indicate which Bell-state was heralded to verify that the phase shifter was operated correctly, and to control shutters (not shown in the setup) that are used to protect the single photon detectors when classical light is being used for AFC preparation.

D Bell-state measurement

At Bob's side, in addition to the telecom qubits source, we placed the Bell-state measurement (BSM) set-up. Our BSM consists of first interfering the qubit to be teleported and the entangled idler photon at a fibre beam splitter (BS)

Supplementary table 2. Possible Bell-state measurement outcomes after detections at the outputs D_1 and D_2 of the beam splitter.

Heralded state	Time bin	D_1	D_2	D_1	D_2
$\|\Psi^-\rangle$	$\|e\rangle$	✓	-	-	✓
	$\|l\rangle$	-	✓	✓	-
$\|\Psi^+\rangle$	$\|e\rangle$	✓	-	-	✓
	$\|l\rangle$	✓	-	-	✓

and later, measuring the photons in the time-bin basis after the BS. A fibre-based polarization controller is used to ensure polarization indistinguishability between them. We connect the two outputs of the fibre BS to superconducting nanowires (IDQuantique ID281, 80 % of detection efficiency, 10 Hz dark count rate).

The triggers of the detections are sent to a time-to-digital converter (TDC) card where they are recorded and further processed to discriminate between the possible results of the BSM in real time. In supplementary table 2 we show the possible combinations of clicks and how we relate them with the outcome of the Bell-state measurement. With our set-up we are only able to distinguish between $|\Psi^+_{2,3}\rangle$ and $|\Psi^-_{2,3}\rangle$ events, limiting the BSM efficiency to the 50% expected for detection set-ups not involving non-linear phenomena [7].

Depending on the experimental configuration (short distance or 1 km of distance) the electronic delay between the detectors and the TDC card varies (from no delay to 5 μs of delay, corresponding to 1 km of distance assuming propagation of classical signals through optical fibres).

E Quantum memory

We use Pr^{3+}:Y_2SiO_5 crystals (Scientific Materials) for the QM as well as the qubit analyzer, both with a doping of 0.05 % Pr^{3+} ions, and dimensions of $2 \times 3 \times 5$ mm, corresponding to the D_1, D_2 and b refractive index axes of the crystals, respectively. The crystals are cooled to approximately 2.7 K in the same closed-cycle cryostat (Optistat AC-V14, Oxford Instruments). All incident light is polarised along the D_2 axis and propagated along the b axis, and we only address Pr^{3+} ions in the crystallographic site 1. The signal photons are sent to the QM crystal where they are stored for a fixed storage time τ_{AFC}. The retrieved photons are then sent to a phase shifter, and finally to the signal interferometer crystal before being detected with a single photon detector (COUNT-10C-FC, Laser Components). An additional beam path is used for each of the crystals to prepare spectral structures in the optical absorption line.

We prepare the AFC structure following the same procedures as in Ref. [2]. To summarise, in the case of the QM, after preparing a 4 MHz wide single-class spectral feature on the $1/2_g$ to $3/2_e$ transition, we send pulses with an amplitude profile which is the Fourier transform of the ideal comb structure. For the qubit analyzer, we do not prepare a single class feature, but instead we prepare an AFC by burning individual spectral holes into the absorption line, resulting an approximately 10 MHz wide structure.

We use two different QM storage times τ_{AFC} in the experiment: 10 μs and 17.5 μs. The resulting storage efficiencies, as measured using single photons from the source at Alice as the input, are 18.8(5) % and 12.2(4) % respectively.

F Phase shift

As explained in Supplementary note 1, the qubit state at the QM after a BSM is not always the same. In general there will be a bit flip and/or a phase flip difference between the input qubit and the teleported qubit (see equation 1). In our case, as we can only discriminate between the Bell-states $|\Psi^+_{2,3}\rangle$ and $|\Psi^-_{2,3}\rangle$, the teleported qubits will be π-shifted with respect to each other.

Thanks to the storage time of our QM, Alice still has access to the qubit by the time she learns the result of the BSM; this is why we can make a unitary operation to shift by π the relative phase between the early and late time-bins of the qubit when measuring a $|\Psi^-_{2,3}\rangle$ Bell-state. Therefore, we place a phase shifter (Leysop: EM6X20A-PM-HHT-AR400-700) right after the QM crystal. By driving it with either 0 V (no phase shift) or 2.35 kV (π-phase shift) we can always have the same state before the qubit analyzer. Note that the phase shift has to happen in between the early and late time bins with a precision of $\sim$100 ns. For this task the voltage supplier of the phase shifter has <25 ns rise/fall time (QBU-BT-3012-LJ-SP). Moreover, we fine tune the time when the shift is applied. We prepare the state $|R\rangle$ for teleportation and set the analyzer as $\langle R|$. We only look for events where the BSM projects the telecom qubits joint state into $|\Psi^-_{2,3}\rangle$. With this set-up we maximize the heralded events detected after the analyzer by varying the

time at which we make the phase shift. Going from a minimum of coincidences to a maximum implies that we are correctly transforming the state $1/\sqrt{2}\,(|e\rangle - |l\rangle)$ into $1/\sqrt{2}\,(|e\rangle + |l\rangle)$ as only the second one would be parallel to an analyzer set as $\langle R|$.

In supplementary figure 2 we show this optimization. The y-axis represents the normalized rate of the $\left|\Psi^-_{2,3}\right\rangle$ heralded events going through an analyzer orthogonal to the non-phase shifted qubit and parallel to the π-shifted qubit. The zero in the x-axis has no physical meaning as it corresponds to the time at which we started the optimization. Additionally the brown line corresponds to a numerical model that we use to reproduce the behaviour of the phase shift optimization.

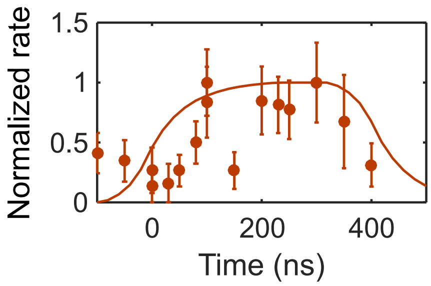

Supplementary figure 2. Adjusting the timing of the phase shifter for $\left|\Psi^-\right\rangle$ events. An input qubit in the state $|R\rangle$ is teleported and only events heralded by a BSM resulting in $\left|\Psi^-\right\rangle$ are considered. The analyzer for the teleported state is set as $\langle R|$. More explanation in the text.

Finally, in supplementary figure 3 we illustrate the action of the phase shifter through two examples.

- For the first two histograms we teleport an input qubit $|+\rangle$ that we send through an analyzer set as $\langle -|$. As we do not apply any phase shift, the state heralded by a $|\Psi^-\rangle$ Bell-state becomes parallel to the analyzer while that heralded by a $|\Psi^+\rangle$ is orthogonal.

- For the last two histograms we teleport $|+\rangle$ and we send it through an analyzer set as $\langle +|$. We switch on the phase shifter only when the heralding Bell-state is $|\Psi^-\rangle$. As a consequence, we see that for any outcome of the BSM the events distribute evenly as both are aligned with the analyzer.

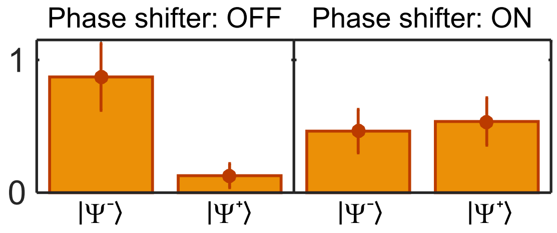

Supplementary figure 3. Comparison between the phase shifter being completely OFF versus ON. The y-axis is normalized to the sum of $\left|\Psi^-\right\rangle$ and $\left|\Psi^+\right\rangle$ events such that the histograms show the relative distribution of events among the different heralding possibilities.

G Experimental sequence

In this section we are going to explain how a generic experimental sequence works for our experiments. The procedure is the following:

- Probabilistic generation of entangled pairs: At Alice, entangled photon pairs are generated probabilistically by a source pumped by a continuous wave laser.
- Unconditional storage: The temporally multimode QM unconditionally stores the signal photons forwarded from the source as a collective excitation of Pr^{3+} ions. The storage time is 10 μs for the short distance experiment and 17.5 μs for the 1 km experiment.
- Teleportation trials: At Bob, we make teleportation attempts every 4.1 μs (this period only changes for the measurement shown in figure 3c of the main text). This implies modulating the OPO light to generate the qubit state that beforehand we chose to teleport. We send these qubits to a BS where they can interfere with the idler photons coming from Alice.

These three last steps are running simultaneously until we get a successful BSM.

- Bell-state measurement: Once the logic control described in Supplementary note 2 D identifies a BSM, we know that we have successfully heralded a teleportation event into the QM. At this point, we stop performing teleportation attempts and we process the detection events to identify which Bell-state we have heralded the teleportation with.
- Phase shift: As explained in Supplementary note 2 F, we only apply 2.35 kV to the phase shifter when the heralding Bell-state is $|\Psi^-\rangle$. The time at which this shift is applied has to be carefully chosen such that it only affects one of the time bins while they are being retrieved from the QM.
- Analysis and detection: Finally, we send our teleported states into the qubit analyzer to quantify their fidelity after the protocol has been completed. We use an SPD to detect the analyzed qubits.
- We once again begin to perform teleportation trials.

Supplementary note 3 EXPECTED FIDELITY

In this section we will discuss the main factors that limit the fidelity that we measured and quantify its expected value ($\mathcal{F}$). Note that most of the subsections will quantify these limitations using the visibility as the figure of merit. For instance, when we teleport states on the equator of the Bloch-sphere, $V_{|x\rangle} = \frac{C_{\langle x|x\rangle} - C_{\langle y|x\rangle}}{C_{\langle x|x\rangle} + C_{\langle y|x\rangle}}$ where C corresponds to the coincidences after teleporting the state $|x\rangle$ and analyze it by measuring in the basis $\langle x|$ or $\langle y|$ ($\langle x|$ and $\langle y|$ are orthogonal bases).

A State generation

The main limitations in the fidelity come from the preparation of the states:

- **Entangled state preparation:** As previously introduced, the coherence time of the laser used to pump the cSPDC source is ~ 1 μs. This sets a limit of the maximum visibility that the $|e\rangle$ and $|l\rangle$ bins can have when interfered. This visibility corresponds to 91.3(7) % and it was estimated by sending the DFG light generated by the cSPDC source when operated as an OPO through a Mach-Zehnder interferometer with the same delay between its paths (420 ns) that we use to define our time bin encoding. Note that a shorter separation in time between bins would not be possible as it would lead to single photon interference effects from one photon overlapping with more than one time bin, because of the long coherence time (120 ns) of the photons generated by the source.
- **Teleported qubit state:** Through a Hong-Ou-Mandel interference measurement between the heralded telecom photon coming from Alice and an attenuated laser pulse coming from Bob, we could estimate the overlap between both sources to be $\eta = 90(5)\%$ using the model introduced in [8]. Here, η takes into account distinguishabilities in all possible degrees of freedom: polarization, temporal shape and frequency.

Note that these limitations will only affect the states prepared on the equator of the Bloch-Sphere. The states prepared on the poles of the Bloch-Sphere are not in a superposition between the $|e\rangle$ and $|l\rangle$ bins of the encoding base. Therefore, we do not expect any limitation due to the limited linewidth of the pump laser or the indistinguishability between qubits at the BSM set-up.

B Pr^{3+}-based time-bin analyzer

The way we analyze the teleported qubit state also limits the maximum fidelity that we can measure. Thus we have to differentiate the two kinds of qubit analyzers that we employed:

- **Analyzer for equatorial states:** As explained in the main text, when we analyzed the states on the equator of the Bloch-sphere we used a second Pr^{3+} doped crystal. We balanced the probability of light being transmitted through or stored in a 420 ns AFC in the crystal. We had previously characterized a very similar technique in the *Supplementary Information* of [2]. In order to estimate the intrinsic visibility limitation that we expect by measuring with this method, we sent pulses of classical light shaped to emulate the temporal profile of our photons. We obtained a value of $V_{analyzer} = 98(1)$ %.

- **Analyzer for the poles:** In order to estimate the fidelity of the poles we prepare a transparency window of 16 MHz in the second Pr^{3+}crystal. When we look at the coincidence histogram between the heralding events at the BSM set-up and the signal photons after being retrieved by the QM and we compare the correct temporal windows, we can quantify the visibility of the teleported state. In supplementary figure 4a in the top (bottom) panel we show an example of how the teleportation of the state $|\phi_3\rangle = |l\rangle$ ($|\phi_3\rangle = |e\rangle$) would look like without considering any non-ideal behaviour. The brown (orange) temporal windows show where the $|e\rangle$ ($|l\rangle$) bin of the analyzer would be. The temporal profile of the individual time bins fits inside its own temporal window. Note that, as explained in equation (1), the teleported qubit will be bin-flipped with respect to the input qubit. We can, for example, calculate the visibility of the teleported input state $|e\rangle$ as:

$$V_{|e\rangle} = \frac{C_{\langle l|e\rangle} - C_{\langle e|e\rangle}}{C_{\langle l|e\rangle} + C_{\langle e|e\rangle}} \tag{2}$$

  where C represents the number of events at each temporal bin. However, the temporal shape of our photons after the storage in the AFC memory will look more similar to those represented in supplementary figure 4b. This is due to the long coherence time of the photons generated at the source with an additional increase because the photon is slightly wider in frequency than the AFC. Here, we can see that the temporal distance between the $|e\rangle$ and $|l\rangle$ bins is not enough to completely avoid leakage from the wings of the other temporal mode. For the coherence time of the stored photons, we quantify this limitation as $\mathrm{V}_{analyzer} = 76$ % when the input state is $|e\rangle$ and $\mathrm{V}_{analyzer} = 64$ % when the input state is $|l\rangle$.

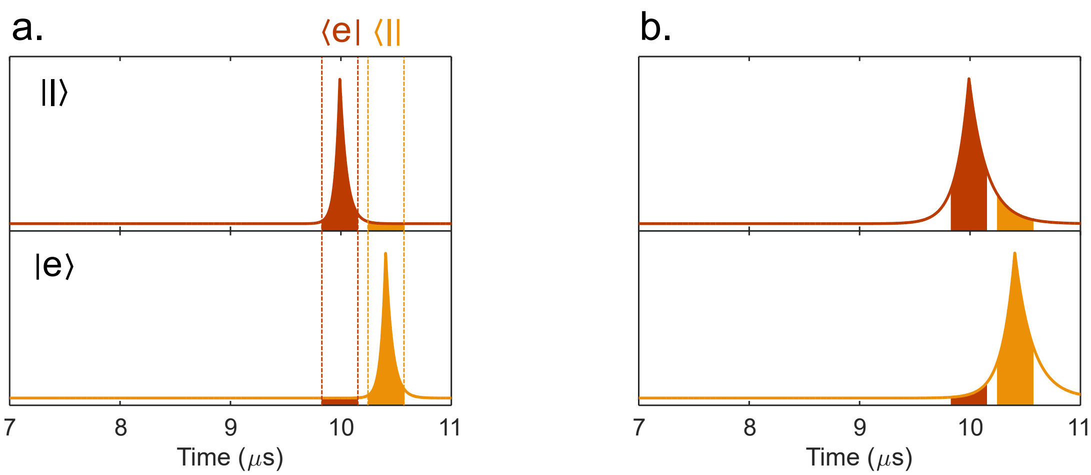

Supplementary figure 4. Examples to represent the way we analyze the poles of the Bloch Sphere. a. Ideal case where the temporal profile of the individual time bins fits inside its own temporal window. b. Non-ideal scenario where one of the wings of the temporal modes falls into another time bin window.

C Statistics of a weak coherent input qubit

In the Supplementary Information of [9], section 5, the authors introduce an analytical model to estimate the fidelity limitations depending on the mean photon number used in the input qubits and the probability of generating an entangled photon pair at their SPDC source. In our case the source of teleported qubits is an attenuated laser, and the source of entanglement is based on an SPDC process. This matches with the same system that this model describes. Therefore, we can use it to estimate the expected fidelity of the equator qubits as:

$$F_{eq} = \frac{1}{2}\left(1 + V_{pump} \cdot \eta \cdot V_{analyzer} \frac{P(1,1,1) + P(1,1,2)}{P(1,1,1) + P(1,1,2) + P(0,2,2) + P(2,0,1)}\right) \tag{3}$$

where $P(i,j,k)$ are the probabilities of having i photons for the input qubit mode at the detector, j photons for the idler mode at the detector and k photons for the signal mode at the detector. For example, it could happen that the BSM is triggered by two photon detections coming from the input qubit ($i = 2$), while the idler photon is lost on its way to the BS ($j = 0$) and the signal photon is detected ($k = 1$). In this case, the state of the input qubit would not be teleported. However, experimentally, we would not be able to distinguish it from the scenario with one photon at each mode ($i = j = k = 1$). If we substitute using the values of our set-up together with all the previous visibilities that we listed, we get $\mathcal{F}_{eq} = 84(2)$ %.

Additionally, the fidelity at the poles will not be affected by coherence limitations nor indistinguishabilities, but it will be affected by $V_{analyzer}$, therefore we can express it as:

$$F_{poles} = \frac{1}{2}\left[\left(2 \cdot \frac{P(1,1,1) + P(1,1,2) + 0.5P(0,2,2)}{P(1,1,1) + P(1,1,2) + P(0,2,2) + P(2,0,1)} - 1\right) V_{analyzer} + 1\right] \tag{4}$$

Taking the average between the values of $\mathrm{V}_{analyzer}$ for $|e\rangle$ and $|l\rangle$ we get an expected $\mathcal{F}_{poles} = 79$ %. Finally, we can estimate the mean fidelity of an arbitrary qubit as $\mathcal{F} = \frac{1}{3}\mathcal{F}_{poles} + \frac{2}{3}\mathcal{F}_{eq}$ resulting in $\mathcal{F} = 82(1)$ %. This value is in good agreement with the fidelity measured experimentally of 85(4) %.

[1] Seri, A. *et al.* Laser-written integrated platform for quantum storage of heralded single photons. *Optica* **5**, 934 (2018).

[2] Rakonjac, J. V. *et al.* Entanglement between a telecom photon and an on-demand multimode solid-state quantum memory. *Physical Review Letters* **127**, 210502 (2021).

[3] Lago-Rivera, D., Grandi, S., Rakonjac, J. V., Seri, A. & de Riedmatten, H. Telecom-heralded entanglement between multimode solid-state quantum memories. *Nature* **594**, 37–40 (2021).

[4] Seri, A. *et al.* Quantum Storage of Frequency-Multiplexed Heralded Single Photons. *Physical Review Letters* **123**, 080502 (2019).

[5] Rieländer, D., Lenhard, A., Mazzera, M. & Riedmatten, H. D. Cavity enhanced telecom heralded single photons for spin-wave solid state quantum memories. *New Journal of Physics* **18**, 1–11 (2016).

[6] Rieländer, D. *et al.* Frequency-bin entanglement of ultra-narrow band non-degenerate photon pairs. *Quantum Science and Technology* **3**, 014007 (2017).

[7] Calsamiglia, J. & Lütkenhaus, N. Maximum efficiency of a linear-optical bell-state analyzer. *Applied Physics B* **72**, 67–71 (2001).

[8] Padrón-Brito, A., Lowinski, J., Farrera, P., Theophilo, K. & de Riedmatten, H. Probing the indistinguishability of single photons generated by rydberg atomic ensembles. *Phys. Rev. Research* **3**, 033287 (2021).

[9] Valivarthi, R. *et al.* Quantum teleportation across a metropolitan fibre network. *Nature Photonics* **10**, 676–680 (2016).
